# Targeting the PI3K/AKT/mTOR Signaling Pathway in Lung Cancer: An Update Regarding Potential Drugs and Natural Products

**DOI:** 10.3390/molecules26134100

**Published:** 2021-07-05

**Authors:** Iksen, Sutthaorn Pothongsrisit, Varisa Pongrakhananon

**Affiliations:** 1 Department of Pharmacology and Physiology, Faculty of Pharmaceutical Sciences, Chulalongkorn University, Bangkok 10330, Thailand; ikseniksen08@gmail.com (I.); sutthaorn.po@gmail.com (S.P.); 2 Department of Pharmacy, Sekolah Tinggi Ilmu Kesehatan Senior Medan, Medan 20131, Indonesia; 3 Preclinical Toxicity and Efficacy Assessment of Medicines and Chemicals Research Cluster, Chulalongkorn University, Bangkok 10330, Thailand

**Keywords:** mammalian target of rapamycin (mTOR), natural compounds, protein kinase B (PKB/AKT), phosphatidylinositol-3-kinase (PI3K)

## Abstract

Lung cancer is one of the most common cancers and has a high mortality rate. Due to its high incidence, the clinical management of the disease remains a major challenge. Several reports have documented a relationship between the phosphatidylinositol-3-kinase (PI3K)/ protein kinase B (AKT)/ mammalian target of rapamycin (mTOR) pathway and lung cancer. The recognition of this pathway as a notable therapeutic target in lung cancer is mainly due to its central involvement in the initiation and progression of the disease. Interest in using natural and synthetic medications to target these signaling pathways has increased in recent years, with promising results in vitro, in vivo, and in clinical trials. In this review, we focus on the current understanding of PI3K/AKT/mTOR signaling in tumor development. In addition to the signaling pathway, we highlighted the therapeutic potential of recently developed PI3K/AKT/mTOR inhibitors based on preclinical and clinical trials.

## 1. Introduction

Lung cancer is the most common cancer worldwide and the leading cause of cancer-related death, affecting millions of people every year [1]. Among the common subtypes of lung cancer, non-small cell lung cancer (NSCLC) represents 85% of lung cancer cases [2], while small cell lung cancer (SCLC) represents approximately 15% of all cases of lung cancer [3]. Smoking is the major risk factor for NSCLC, while this disease also affects never-smokers [4]. NSCLC is divided into squamous cell carcinomas, adenocarcinomas and large-cell carcinomas [5]. Squamous cell carcinoma is responsible for 30% of lung cancer cases worldwide and is related to a smoking history. It usually grows in the bronchi that branch off the main left or right bronchus in the center of the chest [6]. Lung adenocarcinoma is responsible for 40% of all lung cancers. Large cell lung carcinoma is the least common type of NSCLC, accounting for approximately 10–15% of cases. Approximately 60% of patients with NSCLC present metastatic disease, with only a 4% 5-year survival rate [7]. The most common metastatic sites for NSCLC are the bone, respiratory tract, adrenal glands, nervous system and liver [8].

The main strategy in the treatment of lung cancer is chemotherapy, which may improve the quality of life of patients [9]. Chemotherapeutic drugs have many benefits in some cases, but they have multiple side effects. These agents are highly toxic to all cells in the body, eliminating both normal and cancer cells [10]. Chemotherapeutic drugs also lead to changes in the normal function of the cells, and fatigue, inflammation, anemia, hair loss and bleeding complications often occur. Furthermore, patients may acquire resistance to chemotherapy, and other combinations of chemotherapeutic drugs must be used for treatment [11]. Other choices for the early stages of lung cancer are surgery and radiation; however, these two choices of treatment have disadvantages, such as a lower efficacy on microscopic cancer cells at the edges of the tumor [12].

As lung cancer treatment increases interest in targeted therapy, systemic chemotherapy is still the standard treatment for lung cancer [13]. Unfortunately, the drug resistance in advanced stage lung cancer limits the success rate of clinical outcome. More than 90% of lung cancer has an intrinsic drug resistance, and the early responders who undergo chemotherapy develop resistance swiftly [14]. The standard therapy for lung cancer includes platinum-based drugs (cisplatin) and taxanes (paclitaxel and docetaxel) [15]. Despite massive clinical progress, drug resistance has limited the therapeutic effectiveness [16,17]. Although the identification of the molecular causes and prediction of biomarkers for chemotherapy sensitivity are important, they need persistent investigation to overcome this problem.

At present, the treatment paradigm for lung cancer has shifted to targeted therapy. This type of treatment attacks specific features of cancer cells known as molecular targets that are either cell surface receptors or intracellular molecules. Many types of targeted therapies for lung cancer are currently available, such as epidermal growth factor receptor (EFGR) inhibitors (erlotinib and gefitinib) or anaplastic lymphoma kinase (ALK) inhibitors (crizotinib and alectinib). The development of specific targeted therapy such as EGFR inhibitors has been shown to increase overall survival (OS) at around 42.6% (from 4.7 to 6.7 months), which shows the benefit of targeting an oncogenic driver compared to the standard first-line chemotherapy [18]. Targeted therapy, on the other hand, does not work if the tumor does not contain the specified target. If the target turns out to be less critical for cancer growth than previously expected, the drug may not be effective. Moreover, cancer cells may develop resistance to targeted therapy, resulting in a poor response [19].

Currently, PI3K/AKT/mTOR signaling has been reported as an emerging source of lung cancer aggressiveness [20]. The development of therapies targeting PI3K/AKT/mTOR signaling is receiving extensive attention from researchers and new drugs continue to be discovered. Several PI3K/AKT/mTOR-targeted therapies, such as buparlisib (PI3K inhibitor), MK2206 (AKT inhibitor), sirolimus (mTOR inhibitor), and perifosine (dual PI3K/AKT inhibitor), are undergoing clinical trials as treatments for lung cancer [21,22,23,24]. Furthermore, several preclinical studies of natural compounds are attracting interest, and their potent inhibitory effects on this signaling pathway have been reported [25,26,27]. As an excellent example, natural compounds with the quinones structure, widely distributed naturally, have attracted enormous attention due to their several mechanisms as an anticancer therapy [28]. The current review will focus on the role of the PI3K/AKT/mTOR pathway in lung cancer aggressiveness and promising drugs/natural compounds targeting this signaling pathway that are undergoing clinical or preclinical trials.

## 2. PI3K/AKT/mTOR Signaling Pathway

**Figure 1:**
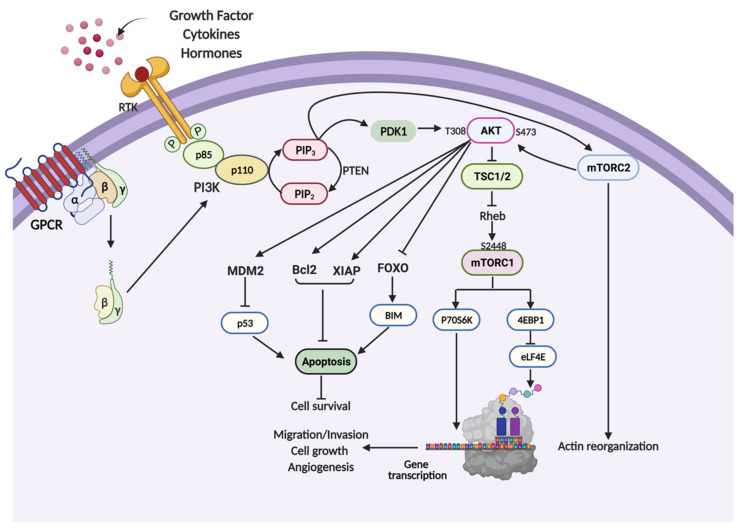
Summary of the phosphoinositide 3-kinase (PI3K)/protein kinase B (AKT)/mammalian target of rapamycin (mTOR) signaling pathway (created with BioRender.com). The PI3K pathway is activated when receptor tyrosine kinases (RTKs) or G protein-coupled receptors (GPCR) bind to growth factors and lead to AKT activation. Upon activation, AKT triggers the phosphorylation of mTOR, p70S6 kinase 1 (p70S6K) and eukaryotic translation factor 4E-binding protein 1 (4EBP1), resulting in the modulation of gene transcription related to cancer aggressiveness. AKT also stimulates cell death resistance by upregulating mouse double minute 2 homolog (MDM2) and prosurvival proteins such as Bcl2 and X-linked inhibitor of apoptosis protein (XIAP) and downregulating Forkhead box O3 (FOXO3) and proapoptotic proteins. mTORC2 phosphorylates AKT and induces actin reorganization, resulting in cell motility.

The protein kinase B (AKT)/mammalian target of rapamycin (mTOR) pathway is initiated by the activation of phosphoinositide 3-kinases (PI3K). PI3K, an enzyme consisting of a large family of lipid and serine/threonine kinases, is normally involved in lipid synthesis [29]. PI3K, a heterodimeric protein, is composed of p110 catalytic and p85 regulatory subunits [30]. It is a downstream effector that is activated in response to a variety of extracellular stimuli, such as hormones, cytokines and growth factors. The binding of growth factors to their cell surface receptors, including G protein-coupled receptor (GPCR) and growth factor/receptor tyrosine kinase (RTK), activates the receptor complex, which induces the dimerization and phosphorylation of PI3K (Figure 1) [31,32,33,34]. The p110 catalytic subunit of PI3K converts phosphatidylinositol 4,5-bisphosphate (PIP2) into phosphatidylinositol 3,4,5-trisphosphate (PIP3). Incidental proteins, known as PIP2 and PIP3, interact with PH domain proteins located on the inner layer of the plasma membrane, resulting in conformational changes in the protein molecules [35]. During activation, PIP3 induces the activation of phosphoinositide-dependent kinase-1 (PDK1) and downstream targets of AKT. Under normal conditions, phosphatase and tensin homolog (PTEN) functions as a tumor suppressor protein that suppresses the activation of AKT [36].

AKT is a serine/threonine kinase that contains a core kinase domain with a threonine residue (T308) and a *C*-terminal tail domain that binds to mTOR complex 2 (mTORC2). Several studies have reported that phosphorylated AKT (p-AKT) promotes aggressive cancer behaviors such as cell proliferation, invasion, metastasis, and angiogenesis, and prevents programmed cell death through the regulation of several downstream effectors [37,38,39,40]. Three different isoforms of AKT have been identified: AKT1, AKT2 and AKT3. Activating phosphorylation of the AKT protein at Ser473 and Thr308 further induces the phosphorylation of tuberous sclerosis complex 1/2 (TSC1/2) to inhibit its function; as a result, Rheb activates mTORC1 [41,42,43].

Overexpression of mTOR is commonly observed in various types of cancers [44]. mTOR forms two types of complexes, namely, mTORC1 and mTORC2 [45]. Each complex shares similarities in the mTOR kinase (central catalytic component), mLST8 (scaffolding protein), DEPTOR (mTOR regulatory subunit) and Tti1/Tel2 complex (complex assembly and stability of mTOR) domains. mTORC1 contains Raptor and PRAS40, while mTORC2 contains Rictor and mSin1. In contrast to mTORC1, mTORC2 regulates AKT, which is activated by direct signal transduction from PDK1 [46].

The PI3K/AKT/mTOR signaling pathway is engaged in a wide spectrum of metabolic processes in which it monitors energy, nutrient and stress levels. Transcription of genes associated with the cancer promotor stimulated by mTORC1 occurs through the phosphorylation of its downstream effectors eukaryotic translation factor 4E-binding protein 1 (4EBP1) and p70S6 kinase 1 (S6K1) [44]. They regulate translational initiation by upregulating the expression of positive regulators of mRNA translation, which are required for the S phase of the cell cycle. mTORC2 phosphorylates AKT and serine/threonine protein kinase 1 (SGK1) at the C-terminus, which regulates remodeling of the actin cytoskeleton [47].

Deregulation of the PI3K/AKT/mTOR signaling pathway by either the mutation or amplification of genes involved in the PI3K pathway, loss of the tumor suppressor PTEN, or overactivation of RTKs, has been observed in various cancer cells, contributing to tumor progression and metastasis [48,49,50,51]. *PIK3CA*, the gene encoding the catalytic subunit of the PI3Kα isoform, is frequently mutated in various human cancers, including breast, ovarian and lung cancers [52,53,54]. Two hotspot E542K and E545K mutations in the p110 subunit alter the conformation of PI3Kα, in which an active site of PI3Kα is exposed at the membrane and is subsequently activated [55,56]. The H1047R mutation located in the kinase domain is able to mimic Ras action, which induces the membrane localization of PI3K [57].

Mutation or loss of PTEN, a negative regulator of the PI3K signaling pathway, has also been observed in many cancers [58,59]. PTEN mutations frequently occur in the phosphatase domain, which impairs its tumor suppressor activity [59,60]. Furthermore, the mutation of AKT itself also increases AKT activity in cancer cells. The E17K mutation in the PH domain of AKT enhances the binding of AKT to PIP3, leading to AKT phosphorylation [61]. EGFR, an upstream regulator of the PI3K signaling pathway, is commonly mutated and/or overexpressed in various cancers. Missense mutations and in-frame deletions in EGFR have been found to autoactivate downstream targets, including the PI3K signaling pathway [62].

## 3. PI3K/AKT/mTOR Pathway in Cell Survival and Chemotherapeutic Resistance

Cell survival and chemotherapeutic resistance are inseparable from the PI3K/AKT/mTOR signaling pathway. Several factors related to this signaling pathway include the Bcl-2 family, X-linked inhibitor of apoptosis protein (XIAP), mouse double minute 2 homolog (MDM-2) and Forkhead box O3 (FOXO3a) transcription factor (Figure 1) [63,64,65,66,67]. Based on accumulating evidence, the PI3K/AKT/mTOR signaling pathway is abnormally activated in many cancers, causing apoptosis deregulation and chemotherapeutic resistance [68]. In an apoptosis process, the Bcl-2 protein family is the main factor contributing to cancer survival and multidrug resistance [69]. Bcl-2 family proteins alter the permeability of the mitochondrial membrane, which leads to the release of cytochrome C and caspase activation-mediated cell death. Overexpression of components of the AKT signaling pathway disturbs the balance of Bcl-2 family proteins. AKT itself induces the phosphorylation of BAD (Bcl-2-associated agonist of cell death) at Ser136 or Ser112, resulting in the disruption of heterodimerization of prosurvival Bcl-2 proteins such as Bcl-xL and Bcl-2 and subsequently prevents apoptosis [70,71].

Several studies have shown that XIAP is regulated by AKT signaling [55,64,65]. Activated XIAP directly binds to and inhibits caspase activity; as a result, the apoptosis process is suppressed. According to Dan et al., AKT stabilizes XIAP at the Ser87 amino acid by phosphorylation, and this mechanism is able to inhibit cisplatin-induced cell death [63,72,73]. MDM-2, a negative regulator of the tumor suppressor p53, is also phosphorylated by AKT at Ser166 and Ser186. Phosphorylated MDM-2 binds to and blocks the *N*-terminal transactivation domain of p53, which mediates p53 degradation by the ubiquitination process [74,75]. The degradation of p53 disturbs the balance between prosurvival and proapoptotic proteins, since p53 is responsible for proapoptotic BAX transcription [76,77]. In contrast, a loss of p53 induces resistance to apoptosis mediated by chemotherapeutic agents [64,65].

The FOXO3 protein is a FOXO transcription factor and a member of a subgroup of the Forkhead family. FOXO3 promotes apoptosis signaling by either inducing the expression of multiple proapoptotic members of the Bcl-2 family proteins or stimulating the expression of death receptor ligands such as Fas ligand and tumor necrosis factor-related apoptosis-inducing ligand (TRAIL) [78]. FOXO3 is phosphorylated and is transported out of the nucleus through an AKT-dependent mechanism [78,79]. Overexpression of AKT also inhibits the expression of the FOXO3 transcription factor [78,80].

## 4. PI3K/AKT/mTOR Pathway in Cell Proliferation

Activated AKT/mTOR are considered important key elements that regulate cell proliferation [81]. Several inhibitors of mTOR, either chemical agents such as rapamycin or nutrient starvation, induce cell cycle arrest in the G1 phase [82]. Downstream effectors of mTOR, such as 4E-BP1 and P70S6K, are required for G1 phase progression through the transcriptional regulation of G1 cyclins (D- and E-type cyclins) or the cytoplasmic sequestration of cyclin-dependent kinase inhibitor 1 (p21^CIP1/WAF1^) and cyclin-dependent kinase inhibitor 1B (p27^Kip1^), which inhibit these cyclin kinase inhibitors [82,83]. mTOR also facilitates the binding of cyclin D1 to cyclin-dependent kinase (CDK) to initiate cell division. Overexpression of cyclin D1 induces the cell cycle transition from the G1 to the S phase. In addition, mTOR plays a key role in controlling the synthesis of biological macromolecules such as proteins, nucleotides and lipids that are necessary for cell growth [82].

## 5. PI3K/AKT/mTOR Pathway in Cancer Cell Metastasis

Cancer metastasis is the process by which cancer cells dissociate, migrate, and invade other sites. The PI3K/AKT/mTOR pathway participates in cancer metastasis in which cancer cells are stimulated by the activation of RTKs, cytokines, or hormones. Active AKT induces phosphorylation of mTORC1 and its downstream targets, such as 4EBP1 (Thr 37/46) and p70S6K (Thr 389). Activation of 4EBP1 induces the translation of several transcription factors, such as Snail, Slug, and Twist [84,85,86]. Activation of these transcription factors leads to an upregulation of epithelial-mesenchymal transition (EMT) markers (vimentin and N-cadherin) and a decrease in the expression of epithelial markers (E-cadherin, ZO-1, and claudin) [87,88].

According to previous findings, the PI3K/AKT/mTOR pathway governs cancer cell migration and invasion by regulating F-actin reorganization [89]. AKT activates palladin, an actin-associated protein, by phosphorylating Ser507 to regulate cell migration [90]. Activated p70S6K, which is mediated by the PI3K/AKT/mTOR axis, functions as an upstream regulator of Rac1 and Cdc42 that controls actin reorganization during cancer cell movement [91,92]. Activated p70S6K directly interacts with cross-linked F-actin to prevent actin depolymerization by cofilin family proteins [91]. The loss of mTOR activity induced by Akt inhibition contributes to a disruption of protrusive structure formation (lamellipodia and filopodia) and F-actin organization [93]. In addition, p70S6K is also involved in the expression and activity of matrix metalloproteinases (MMPs), proteolytic enzymes that are responsible for extracellular matrix degradation during cell invasion [94]. Activation of p70S6K promotes MMP-9 mRNA expression and stimulates proteolytic activity in ovarian cancer cells [95]. Moreover, knockdown of AKT expression, which leads to a loss of functional mTOR and p70S6K, results in MMP-2 and MMP-9 mRNA downregulation in lung cancer cells [96]. In addition, phospho-eIF4E induces the translation of MMP-3 and MMP-9 [88,97]. Based on these findings, the PI3K/AKT/mTOR signaling pathway plays a pivotal role in cancer cell migration and invasion, and the blockade of molecules in this pathway represents a potential approach for cancer treatment.

## 6. PI3K/AKT/mTOR Pathway in Cancer Angiogenesis

Angiogenesis or the development of new blood vessels is one of the common hallmarks of cancer and is crucial for cancer progression, development and metastasis. This process is necessary to supply nutrients and oxygen to compensate for rapid tumor growth [98,99]. Tumor blood vessel growth is initiated by hypoxia-mediated upregulation of both the hypoxia-inducible factor-1α (HIF-1α) mRNA and protein. In a normal oxygen environment, HIF-1α undergoes ubiquitination through activation of hydroxylation by prolyl hydroxylase domain proteins (PHDs) at residues Pro 402 and 564. Under hypoxic conditions, the level of HIF-1α is stabilized as a result of PHD inhibition, subsequently causing an accumulation of the HIF-1α protein [100,101,102]. HIF-1α forms a complex with HIF-1β and activates the transcription factor hypoxia response element (HRE), which induces the transcription of several proangiogenic factors, such as MMPs, vascular endothelial growth factor (VEGF), angiopoietin-1/2 and nitric oxide synthase (NOS) [103]. Several studies have shown that a high level of HIF-1α activates MMP transcription, which is crucial for the degradation of extracellular matrix (ECM) and connective tissue barriers and is necessary for proangiogenic factors to reach endothelial cells [104,105]. HIF-1α also induces the production of VEGF, a secretory cytokine, leading to the growth of endothelial cells [106].

HIF-1α was reported to be regulated by the PI3K/AKT/mTOR signaling pathway. AKT/mTOR induces downstream signaling, such as 4EBP1, which is essential for inhibiting cap-dependent mRNA translation and increasing the translation of the HIF-1α transcription factor [101]. In addition to its effects on HIF-1α activity, AKT induces angiogenesis by promoting cell motility and invasion in NSCLC [101]. The overactivation of AKT alters the distribution of endothelial nitric oxide synthase 3 (eNOS), which leads to an accumulation of nitric oxide (NO) and the remodeling and formation of blood vessels [40]. Furthermore, the suppression of AKT/mTOR/p70S6K signaling is reported to attenuate endothelial cell proliferation, which is critical for controlling the tumor microenvironment and angiogenesis [107,108].

## 7. Current Research on PI3K/AKT/mTOR Inhibitors in Lung Cancer

Due to the substantial increase in the number of new therapeutic agents that target specific molecular pathways, a higher degree of biochemical precision in therapeutic drugs can now be achieved than were previously available with conventional chemotherapeutic drugs. Since the PI3K/AKT/mTOR pathway is considered a potential target for anticancer drug research and development, numerous potent molecules or combinations are undergoing clinical trials. This review summarizes several drugs targeting PI3K/AKT/mTOR signaling pathways that are being investigated in various phases of clinical trials (Table 1).

### 7.1. PI3K Inhibitors

**Figure 2:**
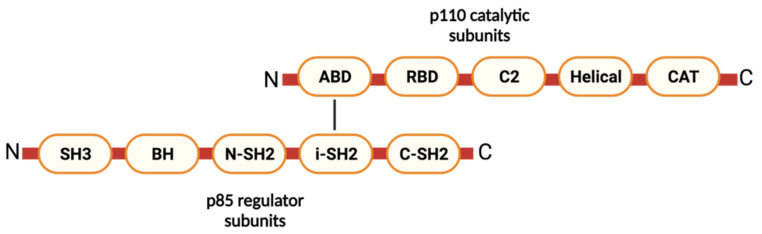
Structure of class IA PI3K (created with BioRender.com). Each class IA PI3K consists of p110 catalytic and p85 regulatory subunits.

The PI3K family is divided into four groups (classes I, II, III and IV) according to their structure, regulation and substrate specificity [109]. Class I PI3Ks are most widely reported to play a key role in the regulation of tumor progression and metastasis, providing important therapeutic targets [109]. Class I PI3Ks are further classified into two subclasses, namely, subclass IA (PI3Kα, β and δ) and subclass IB (PI3Kγ) [109]. Class IA enzymes are heterodimeric molecules containing p110 catalytic and p85 regulatory subunits (Figure 2). Five domains of p110 subunits are present in class IA enzymes, including an adaptor binding domain (ABD), a Ras-binding domain (RBD), a C2 domain, a helical domain and a catalytic kinase domain (CAT). The p85 subunit contains five domains: an *N*-terminal SH3 domain, a Rho-GAP domain, an nSH2 domain, an iSH2 domain and a cSH2 domain [110,111]. In the basal state, p110 and p85 subunits form a complex via the interaction of four domains (ABD-CAT domain, ABD-iSH2 domain, and helical-nSH2 domain), and part of the RBD domain of the p110 subunit is locked in the ATP binding site of the neighboring kinase domain, resulting in the inhibition of enzyme activity [112]. Class IB PI3Kγ also shares structural features; however, it does not contain an *N*-terminal p85-binding motif that controls its activity [110].

Class IA PI3Ks are activated in response to activation of RTKs and Ras proteins. The SH2 domain of p85 is released from the complex and binds to the phosphorylated tyrosine motif (pyxxm) in RTKs, and the RBD domain also directly interacts with the active Ras protein (Ras-GTP) to promote membrane localization of PI3K [112]. ATP occupies the pocket site to mediate the phosphorylation of membrane-localized PIP2 to PIP3, a secondary messenger required for the AKT activation or other downstream molecules. Meanwhile, PI3Kγ is activated by G-protein-coupled receptors (GPCRs) and is regulated via heterotrimeric G proteins [113]. Small-molecule inhibitors of PI3K have been developed to target the ATP binding site in the kinase domain, since class I PI3Ks have a highly conserved ATP binding region and have similar three-dimensional structures among PI3K isoforms [111]. The ATP binding site is located between the two lobes of the kinase domain and is separated by a hinge region [114]. The structure of a small molecule mimicking the adenine ring of ATP anchors in the binding site via hydrogen bonds, leading to a disruption of enzyme activity [115]. The presence of the hinge interaction is preserved in most PI3K inhibitors; however, the interaction of small molecules with other regions surrounding the ATP binding site, including the lower hinge, kβ3-kβ4 strands and p-loop region, also contributes to isoform selectivity [114].

Numerous studies indicate that hyperactivity of PI3K signaling is strongly associated with tumor growth, tumor microvessel density and the increased invasive and chemotactic abilities of cancer cells [116]. Currently, several groups of drugs targeting PI3K have been developed, such as selective PI3K inhibitors, pan-PI3K inhibitors and dual PI3K/AKT or PI3K/mTOR inhibitors. Buparlisib (BKM-120) is a selective PI3K inhibitor of p110α, β, δ and γ with IC_50_ values of 52 nM, 166 nM, 116 nM and 262 nM, respectively, in an ATP-competitive manner, thereby inhibiting the activation of the secondary messenger phosphatidylinositol–3,4,5-trisphosphate [117,118]. It is now being investigated in lung cancer either alone or in combination with other agents (NCT01723800, NCT01570296, NCT01911325, and NCT02194049) [21,119,120,121]. The clinical treatment of patients with alterations in the PI3K pathway (mutated or amplified PIK3CA and/or mutated PTEN and/or null/low PTEN protein expression) in advanced solid tumors with buparlisib at a maximum tolerated dose (MTD) of 100 mg/d is safe and well tolerated [122].

Pictilisib or GDC-0941 is a potent inhibitor of PI3Kα/δ with an IC_50_ of 3 nM [123]. In a clinical study of patients with advanced solid tumors, the administration of pictilisib reduced the level of phosphorylated AKT at serine 473 by more than 90% in platelet-rich plasma at 3 h following the administration of the MTD [124]. Pictilisib itself is undergoing clinical trials in patients with advanced NSCLC in combination with several chemotherapies, such as paclitaxel, carboplatin, pemetrexed, cisplatin, and bevacizumab (NCT00974584) [125]. Based on the data, pictilisib can be used safely in combination therapy, with only common adverse events that normally occur in the standard treatment for NSCLC recorded [126].

Idelalisib is an oral competitive inhibitor of the ATP binding site, specifically at the PI3Kδ catalytic domain, with an IC_50_ of 2.5 nM [127]. Idelalisib is the first PI3K inhibitor approved by the US Food and Drug Administration (FDA) for the treatment of lymphoma [128]. Currently, the combination of pembrolizumab and idelalisib is undergoing a clinical trial in patients with NSCLC who do not respond to immunotherapy (NCT03257722) [129].

Alpelisib (BYL719) is a selective PI3Kα inhibitor derived from 2-aminothiazole with an IC_50_ of 5 nM [130,131]. Alpelisib has been approved by the FDA for the treatment of HER2-positive advanced breast cancer with a PIK3CA mutation, which increases the 7.9-month survival rate of patients with advanced breast cancer, according to a phase III clinical trial (NCT04208178) [132,133]. Alpelisib is being investigated as a treatment for advanced solid tumors in combination with MEK162, an inhibitor of MEK (NCT01449058) [134], using the MTD of 200 mg/d [135].

Serabelisib (TAK-117) is a potent and selective oral PI3Kα inhibitor (IC_50_ of 21 nM). A phase I study on dose escalation reported that TAK-117 showed an acceptable safety profile at the intermittent MTD (900 mg). Observations of grade ≥3 drug-related ALT/AST elevations were lower after the intermittent treatment than after daily treatment with TAK-117 [136]. A further clinical trial of serabelisib in combination with canagliflozin in patients with lung cancer is being performed [137].

Taselisib (GDC-0032) is a potent and selective inhibitor of class I PI3Ks (α, δ, and γ) with IC_50_ values of 0.29, 0.12 nM, and 0.97 nM, respectively, and satisfactory antitumor effects on MCF7 and HER2 xenograft models [138]. Taselisib is being investigated in a clinical trial for several types of advanced or metastatic solid tumor and non-Hodgkin’s lymphoma (NHL), together with hormone receptor-positive breast cancer (NCT01296555) [139]. For lung cancer, a phase II clinical trial (NCT02785913) was conducted in patients with recurrent and stage IV squamous cell lung carcinoma and showed that single-agent treatment with taselisib does not sufficiently improve the overall survival rate of patients with lung cancer, prompting a speculative hypothesis that taselisib may work better in combination with other agents in patients with advanced NSCLC [140].

Gedatolisib (PF05212384) is another type of PI3K inhibitor that also functions as an mTOR inhibitor. Gedatolisib is potent and selective for PI3Kα and PI3Kγ with IC_50_ values of 0.4 and 5.4 nM, respectively [141]. In an in vivo xenograft model of MDA-361 breast cancer cells, gedatolisib induced tumor growth arrest at a dose of more than 10 mg/kg [142]. Due to its promising efficacy, a phase I (NCT02920450) dose escalation trial of gedatolisib is being performed with the combination of paclitaxel and carboplatin in patients with advanced or metastatic NSCLC [143]. Another phase I clinical trial (NCT03065062) of gedatolisib with a CDK4/6 inhibitor (palbociclib) has been ongoing since 2017 in patients with several advanced solid tumors, such as squamous cell lung, pancreatic and head and neck cancers [144].

Voxtalisib (SAR245409/XL765) is a dual-targeting drug that acts by inhibiting the kinase activities of PI3K and mTOR with an IC_50_ of 9 nM for PI3Kγ [145]. An in vitro study conducted in mucinous ovarian cancer cells indicated that pimasertib and voxtalisib exhibit potent synergistic activity [146]; however, their clinical efficacy in a dose-escalation trial of patients with advanced solid tumors, including NSCLC (phase IB), was not sufficient to consider further due to their poor tolerability (NCT01390818) [147].

### 7.2. AKT Inhibitors

**Figure 3:**
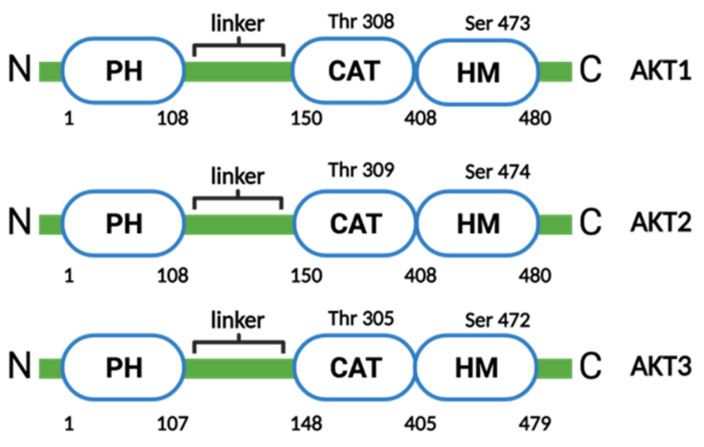
Structure of human AKT family isoforms (created with BioRender.com). Each family member consists of a pleckstrin homology (PH) domain, a catalytic (CAT) domain and a hydrophobic motif (HM).

The AKT kinase family consists of three isoforms: AKT1, AKT2, and AKT3. All AKT isoforms are comprised of three conserved domains: an *N*-terminal pleckstrin homology (PH) domain, a central kinase catalytic (CAT) domain and a *C*-terminal extension (EXT) containing a regulatory hydrophobic motif (HM) (Figure 3). The PH and CAT domains of AKT are connected to the linker region (LINK), which shows no significant homology to other protein kinases [148]. AKT activity is regulated by phosphorylation and dephosphorylation in an Akt conformation-dependent manner. In the absence of stimulation, the PH and CAT domains of AKT are connected by intramolecular interactions to maintain AKT in an inactive state (PH-in conformation) in the cytoplasm [149]. AKT is activated in response to the binding of PIP3 to the PH domain of AKT, which leads to conformational changes in AKT (PH-out conformation) and translocation to the plasma membrane. The PH-out conformation exposes the CAT and regulatory domains, resulting in phosphorylation at two main residues: threonine residues in the activation loop of the CAT domain (Thr308 in AKT1, Thr309 in AKT2 and Thr305 in AKT3) and serine residues in the HM domain (Ser473 in AKT1, Ser474 in AKT2 and Ser472 in AKT2) [150,151]. Moreover, ATP occupies the ATP binding site located in the CAT domain to decelerate the dephosphorylation of AKT, leading to its full activation [152].

The development of small-molecule AKT inhibitors is mainly focused on the inhibition of AKT-mediated phosphorylation. To date, ATP-competitive inhibitors are some of the most common AKT inhibitors and have been shown to strongly suppress AKT activity. These inhibitors directly bind to the ATP binding pocket of active AKT in a PH-out conformation that leads to paradoxical increases in AKT phosphorylation at threonine and serine residues [153]. Hyperphosphorylation of AKT by ATP-competitive inhibitors has been reported to have noncatalytic functions [154]. However, these drugs are poorly selective inhibitors, since the ATP binding site is highly conserved in many protein kinases [148]. Allosteric inhibitors have been developed to improve the selectivity at AKT. They form irreversible intramolecular interactions with residues in AKT located at the linker region to stabilize AKT in the PH-in conformation, preventing AKT phosphorylation [153]. Several studies have revealed that allosteric inhibitors show a greater specificity and fewer side effects, and some of them have been studied in clinical trials, such as MK2206 [155,156].

MK2206 is an AKT inhibitor that targets all three isoforms of AKT (AKT1, AKT2, and AKT3) with IC_50_ values of 8, 12, and 65 nM, respectively [157]. According to Hirai et al., a combination of MK2206 (120 mg/kg/d) with carboplatin (50 mg/kg/d) or gemcitabine (100 mg/kg/d) inhibits tumor growth in an NSCLC-H460 xenograft model [158]. In a phase I clinical trial for advanced solid tumors, MK2206 was associated with stable disease in patients with lung cancer, in which AKT phosphorylation at S473 was decreased in all tumor biopsies assessed [159]. A combination of MK2206 and docetaxel, carboplatin, and paclitaxel or erlotinib was well tolerated in all tested regimens (NCT00848718) [160,161]. MK2206 was administered as a second-line therapy to patients with advanced NSCLC accompanied by brain metastases, and the data showed that patients with EGFR mutations have a longer median progression-free survival (PFS) than those with wild-type EGFR (15.2 months vs 4.4 months/NCT00663689) [162]. The additional treatment with MK2206 also increased the responsiveness of patients with erlotinib-resistant NSCLC (NCT01294306) [22,156]. A similar trial of MEK2206 in patients with NSCLC who were nonresponsive to prior chemotherapy and gefitinib is being conducted in Taiwan [163].

Capivasertib (AZD5363) is similar to MEK2206, as it inhibits all isoforms of AKT (IC_50_ of 3 nM for AKT1 and 8 nM for AKT2 and AKT3) [164]. A phase I study using dose escalation of capivasertib and enzalutamide in patients with prostate cancer showed that a 400 mg (twice daily) treatment increased the responsiveness in patients with PTEN loss or upregulation of mutations in AKT with a good tolerance limit [165]. Currently, capivasertib is undergoing a phase II clinical trial in patients with NSCLC [166].

Uprosertib (GSK2141795) is an oral ATP-competitive AKT inhibitor with IC_50_ values of 180, 328, and 38 nM for AKT1, AKT2, and AKT3, respectively [145,167]. An early clinical trial of uprosertib in combination with trametinib documented a high incidence of vomiting as an adverse effect, leading to dose interruptions. Approximately 60% of patients receiving this combination develop grade 3 side effects, and thus the clinical trial terminated at an early stage due to fewer pharmacological benefits [168]. An additional clinical trial examining combinations of uprosertib, trametinib, and dabrafenib was being patients in patients with stage IIIC-IV lung cancer [169].

Perifosine is an alkyl-phospholipid that functions as a dual PI3K/AKT inhibitor, which is being investigated in a phase I trial in patients with NSCLC (NCT00399789). This clinical trial aimed to determine the MTD of perifosine that is tolerated in the gastrointestinal tract. Patients with NSCLC will receive daily or weekly doses of perifosine (150 mg/900 mg) [24,170]. Apart from lung cancer, perifosine has also been investigated in colorectal cancer in combination with capecitabine, but failed to undergo a phase 3 clinical trial due to the lack of improvement in the overall survival of the patients [171].

Aspirin is a nonsteroidal anti-inflammatory drug (NSAID). In vitro and in vivo experiments showed that additional treatment with aspirin sensitizes NSCLC cells to osimertinib through a Bim-mediated apoptosis induction [172]. In individuals with colorectal cancer carrying a PI3KCA mutation, treatment with aspirin suppresses the proliferation and decreases p-4EBP1 and p-S6K1 levels. In addition to the downstream effect of aspirin, the activation of upstream pathways, such as PI3K, AKT, mTOR, and Raptor, is also decreased [173,174]. Currently, aspirin is being investigated in a phase I clinical trial with osimertinib, an EGFR inhibitor, in patients with osimertinib-resistant NSCLC (NCT03543683). According to a retrospective analysis of 45 patients with NSCLC in Daping Hospital (China), the patients who received aspirin and osimertinib showed a significantly prolonged median progression-free survival compared to patients treated with osimertinib alone [172,175].

### 7.3. mTOR Inhibitors

**Figure 4:**
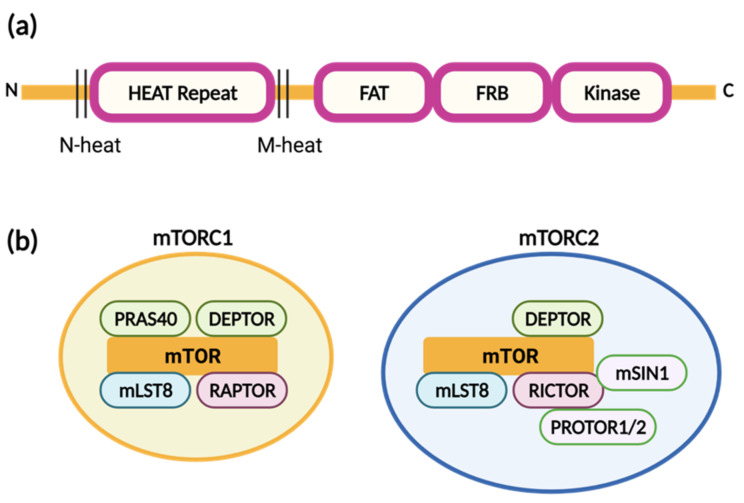
(**a**) Structures of mTOR1 and mTOR2 (created with BioRender.com). Each mTOR consists of HEAT repeats, FAT, FRB, and kinase domains. (**b**) mTOR complex 1 (mTORC1) is composed of mTOR, Raptor, mLST8, Deptor, and PRAS40, whereas mTORC2 includes mTOR, Rictor, mLST8, Deptor, mSin1, and Protor 1/2.

The mTOR kinase family is composed of three functional molecules, mTOR1, mTOR2 and mTOR3, according to the components of the complex, signaling inputs and downstream targets [176]. However, only mTOR1 and mTOR2 have been reported to be closely associated with cancer [46]. Both mTOR1 and mTOR2 molecules consist of two main core subunits: HEAT repeats (NH_2_-terminat HEAT (N-HEAT), middle HEAT (M-HEAT)), FAT, FRB, and kinase domains (Figure 4a) [176,177]. Each mTOR protein interacts with different molecules in a distinct complex. mTOR complex 1 (mTORC1) includes Raptor, which plays an important role in mTORC1 assembly and stability, and substrate identification; mLST8, which stabilizes the complex and phosphorylates the substrate; and PRAS40 and Deptor, which negatively regulate mTORC1 [178]. In addition to mSLT8 and Deptor, mTORC2 contains Rictor, which regulates substrate identification, and Proto ½ and mSIN1, which stabilize and enhance mTORC2 activity (Figure 4b).

mTOR inhibitors are a class of drugs that specifically block the activity of mTOR. In general, mTOR inhibitors consist of two main groups, namely, rapamycin and its analog (rapalog) and ATP-competitive mTOR kinase inhibitors [176]. Rapalog forms a complex with FK506-binding protein (FKBP12) and directly interacts with the FRB domain of mTORC1. This interaction limits access to the ATP binding site cleft in mTOR and prevents its phosphorylation [179]. Since rapalog only binds to the mTORC1 complex, ATP-competitive inhibitors have been developed to overcome this limitation [180]. ATP-competitive inhibitors are designed to target ATP binding sites in the catalytic domains of both mTORC1 and mTORC2, leading to the inhibition of mTOR activity [181]. Some ATP-competitive inhibitors function as dual PI3K/mTOR inhibitors due to structural similarities of the catalytic domain among the protein kinases, and show higher potency in anticancer activity [182,183].

As the first-generation mTOR inhibitor, rapamycin is currently being investigated in a clinical trial in patients with NSCLC in several combinations of sunitinib (NCT00555256) and afatinib (NCT00993499) [184,185]. Its combination with afatinib in patients with NSCLC presenting erlotinib or gefitinib resistance showed lower responsiveness, with an increase in adverse effects and poor tolerability [23].

Another drug derived from rapamycin, temsirolimus, has been approved by the FDA for the treatment of advanced renal cell carcinoma. Preclinical treatment with temsirolimus inhibits the proliferation of several NSCLC lines as well as the antitumor activity of NSCLC xenografts [186]. In another phase II clinical trial of patients with NSCLC carrying HER2 mutations, a combination of temsirolimus and neratinib significantly increased the overall survival rate compared to neratinib alone (NCT01827267) [187]. Currently, treatment with temsirolimus alone is being investigated in a clinical trial conducted in patients with stage IIIB or stage IV NSCLC and small cell lung carcinoma (NCT00079235 and NCT00028028) [188,189]. The combination of temsirolimus and standard chemotherapy (pemetrexed) for NSCLC is also being investigated (NCT00921310) [190]. The MTDs of pemetrexed (375 mg/m^2^/day) and temsirolimus (15 mg i.v. weekly) for stable disease were observed in 37.5% of the total patients [191]. The Washington University School of Medicine reported that three patients achieved a partial response, and two out of eight patients with advanced NSCLC being evaluated had a stable disease after treatment with the combination of temsirolimus (15 mg/week) and radiation, indicating that the treatment was well tolerated (NCT00796796) [192,193].

Metformin is the first-line medication for the treatment of type 2 diabetes mellitus, but recently, several studies have also shown that metformin has anticancer properties by inhibiting mTOR. Metformin is effective at reducing the cancer incidence, and it improves the prognosis of patients whose cancer is diagnosed [194]. Previous retrospective and cohort studies showed that metformin administration was associated with a significantly longer overall survival rate in patients with NSCLC [195,196]. Currently, treatment with metformin alone is being examined in a phase II clinical trial for patients with stage I-IIIa NSCLC [197] or in combination with an anti-PD1 inhibitor such as sintilimab [198].

Onatasertib (CC223) is an mTOR inhibitor that binds to the ATP-binding region in the catalytic site of mTOR. A preclinical experiment showed that onatasertib inhibits the proliferation of several NSCLC cell lines, such as A549, H460, and H23 cells, with IC_50_ values of 0.208, 0.2 and 1.039 µM, respectively. An evaluation of onatasertib in a patient-derived lung adenocarcinoma xenograft showed a 47% reduction in tumor growth after treatment with 10 mg/kg/day [199]. A phase I clinical trial (NCT01545947) of onatasertib is being conducted either with or without combination with erlotinib or azacytidine [200]. In addition to onatasertib, other dual mTOR inhibitors, such as sapanisertib (NCT02417701) and vistusertib (NCT03106155), are also undergoing clinical trials in patients with stage IV or recurrent lung cancer [201,202].

**Table 1:** Ongoing clinical trials of several drugs targeting PI3K/AKT/mTOR signaling in lung cancer.

Drugs	Mechanism of Actions	Combination with	Phase	Refs.
Buparlisib (BKM120)	Class I Pan-PI3K inhibitor	Carboplatin and pemetrexed disodium	Phase I	[119]
		Gefitinib	Phase I	[120]
		Docetaxel	Phase I	[21]
		Cisplatin and etoposide	Phase I	[121]
Pictilisib (GDC-0941)	PI3Kα/δ inhibitor	Paclitaxel Carboplatin (with or without bevacizumab) or pemetrexed, cisplatin, and bevacizumab	Phase I	[125]
Idelalisib	PI3Kδ inhibitor	Pembrolizumab	Phase IB/II	[129]
Alpelisib (BYL719)	PI3Kα inhibitor	MEK162	Phase I	[134]
Serabelisib	PI3K inhibitor	Canagliflozin	Phase IB/II	[137]
Taselisib (GDC-0032)	PI3Kα, δ, and γ inhibitor	-	Phase I	[139]
		-	Phase II	[140]
Gedatolisib (PF05212384)	Dual PI3K/mTOR inhibitor	Paclitaxel Carboplatin	Phase I/II	[143]
		Palbociclib	Phase I	[144]
Voxtalisib (SAR245409/XL765)	Dual PI3K/mTOR inhibitor	MSC1936369B (Pimasertib)	Phase I	[147]
MK2206	AKT inhibitor	Erlotinib	Phase II	[162]
		Erlotinib	Phase II	[22,156]
		Standard chemotherapy and erlotinib	Phase I	[163]
		Gefitinib	Phase I	[161]
Capivarsetib (AZD5363)	AKT inhibitor	-	Phase I	[166]
Perifosine	Dual PI3K/AKT inhibitor	-	Phase I/II	[24]
Uprosertib (GSK-2141795)	AKT inhibitor	Trametinib dimethyl sulfoxide	Phase I/II	[169]
Aspirin	Decrease AKT phosphorylation	Osimertinib	Phase I	[175]
Rapamycin	mTORC1 inhibitor	Sunitinib	Phase I	[184]
		Afatinib (BIBW2992)	Phase I	[23,185]
Temsirolimus	mTORC1 inhibitor	Neratinib	Phase II	[187]
		-	Phase II	[188]
		Pemetrexed	Phase I	[189]
		Radiation	Phase I/II	[192]
Metformin	mTOR inhibitor	-	Phase II	[197]
		Sintilimab	Phase II	[198]
Onatasertib (CC223)	Dual mTOR inhibitor	Erlotinib Azacytidine	Phase I	[200]
Sapanisertib	Dual mTOR inhibitor	-	Phase II	[201]
Vistusertib (AZD2014)	Dual mTOR inhibitor	-	Phase II	[202]

## 8. Natural Compounds Targeting the PI3K/AKT/mTOR Pathway in Lung Cancer

Approximately two-thirds of the anticancer drugs currently available are synthetic products that are derived from natural sources [203]. The increasing abundance of natural resources, especially natural compounds, and their functions as potential templates for efficacious analogs and prodrugs, ensures that natural products are still a versatile source of both active and diverse chemicals [204]. On the other hand, few natural products have been developed into clinically effective drugs, and these unique natural compounds can serve as precursors for the chemical preparation of more efficacious analogs and prodrugs [204,205]. Overall, the important roles of natural products in the discovery and development of novel anticancer drugs have been extensively discussed [203]. The following sections describe the recent research and development of natural compounds targeting the PI3K/AKT/mTOR signaling pathway in lung cancer, particularly new natural compounds that are currently in the preclinical stages of development (Table 2).

### 8.1. Bibenzyl

Bibenzyl compounds have been mainly detected in the *Orchidaceae* family, particularly *Dendrobium* species, including 4,5,4′-trihydroxy-3,3′-dimethoxybibenzyl (TDB) and gigantol [206,207]. Several studies have reported the anticancer activities of TDB, such as the induction of apoptotic cell death and the suppression of metastatic behavior in lung cancer [208,209,210]. TBD was isolated from the *Dendrobium* genus of the orchid family, specifically *Dendrobium ellipsophyllum* [211]. TBD belongs to the bibenzyl group, whose main structure consists of a double phenyl ring linked by ethane. In lung cancer, TBD was able to induce cytotoxicity in several NSCLC cell lines, such as H292, H460 and H23 cells, with IC_50_ values ranging from 100–190 µM. TBD upregulated the expression of the tumor suppressor p53 and proapoptotic protein Bax and downregulated the expression of several antiapoptotic proteins, such as Bcl-2 and Mcl-1. Further experiments reported that TDB also significantly diminished the level of p-AKT [208]. As an antimetastatic drug in lung cancer, TBD concentrations of 0.5–5 µM attenuated the migration and invasion of H292 cells and suppressed the activation of AKT and its downstream signaling, such as focal adhesion kinase (FAK), cell division control protein 42 homolog (CDC42), integrins and EMT markers, including snail and vimentin [209,210].

Gigantol, a bibenzyl compound isolated from the *Dendrobium* genus (*Dendrobium draconis*), exhibits potent anticancer activity against NSCLC cell lines (H460 and H292 cells) [212,213,214,215]. Gigantol inhibits the migration of H460 cells at concentrations of 5–20 µM. The antimigratory effect of gigantol is mediated by the downregulation of p-AKT and caveolin-1 (Cav-1), resulting in a decrease in filopodia formation, an actin-rich membrane protrusion [212]. The expression of several EMT markers, especially slug, is significantly reduced via the ubiquitination mechanism [214]. Apart from inhibitory effects on migration, gigantol is able to sensitize H460 lung cancer cells to anoikis, a form of detachment-induced apoptosis [215]. Bioinformatics analysis using the Kyoto Encyclopedia of the Genes and Genomes (KEGG) pathway database, showing that gigantol downregulated two major signaling pathways, namely the PI3K/AKT and JAK/STAT signaling pathways. Pretreatment with gigantol decreased the levels of cancer stem cell markers, including prominin-1 (CD133) and aldehyde dehydrogenase 1 family member 1A (ALDH1A1), in A549, H460, and H292 cells. In an in vivo tumor xenograft study, gigantol significantly retarded tumor growth, which was characterized by a decrease in Ki-67 expression [213]. Therefore, bibenzyl compounds exert potent anticancer effects through AKT inactivation.

### 8.2. Phenanthrene

Phenanthrene, a group of polycyclic aromatic hydrocarbon-containing compounds composed of three connected benzene rings, was identified in the *Orchidaceae* family, especially *Dendrobium* and *Juncaceae* [216]. Phenanthrene exhibits several biological activities, such as antifungal, antimicrobial, anti-inflammatory, and antitumor activities [217]. Each phenanthrene has various levels of potency and pharmacological effects due to its substitution on the benzene ring. Ephemeranthol A is isolated from *Dendrobium infundibulum*. As a promising natural compound, ephemeranthol A exerts anticancer activity by inducing apoptosis through the activation of several apoptosis-related enzymes, such as caspase-3, caspase-9, and poly (ADP-ribose) polymerase (PARP). In addition to apoptosis induction, ephemeranthol A inhibits the migration of lung cancer cells by inactivating AKT and subsequently suppressing the expression of EMT markers such as N-cadherin, vimentin and slug [218].

Cypripedin, a phenanthrene quinone isolated from *Dendrobium densiflorum*, also induces apoptosis and inhibits migration [219,220]. Cypripedin attenuates lung cancer cell migration through an EMT-dependent mechanism. A mechanistic investigation reported that AKT activity was suppressed in response to cypripedin treatment, consequently increasing GSK-3β expression and mediating slug degradation via proteasomal processes [220]. Furthermore, overexpression of constitutively active AKT minimizes the pharmacological activity of cypripedin, indicating that AKT is an important target of its antimetastatic effect.

Likewise, erianthridin is a phenanthrene derived from *Dendrobium formosum* that was recently reported to have potent antimetastatic and cytotoxic effects on lung cancer [96,221]. Erianthridin suppresses the metastatic behavior of A549 and H460 lung cancer cells through the AKT/mTOR pathway. Molecular docking experiments revealed that erianthridin directly binds to an ATP binding site in the protein kinase domain through hydrogen bonding and van der Waals interactions. As a downstream signaling molecule in this pathway, actin stress fiber and lamellipodia formation are gradually decreased, and the expression of the MMP2 and MMP9 mRNAs are extensively reduced in a dose-dependent manner. Furthermore, an in vivo metastasis model confirmed the strong suppressive effect of this compound on lung colonization. Interestingly, erianthridin was not toxic to normal lung and tubular epithelial cells, whereas cytotoxicity was often observed in response to cisplatin, a standard therapy for lung cancer [221]. Overall, phenanthrene derivatives are strong AKT inhibitors, suggesting that they might serve as prototype compounds for further anti-lung cancer research and development.

### 8.3. Phenolic and Flavonoids

Phenolic compounds are characterized by molecules containing at least one hydroxyl group attached to aromatic hydrocarbons. Phenolic compounds exhibit significant benefits in several diseases, mostly through their antioxidant activity [222]. Phoyunnanin E is a natural phenolic compound that is isolated from *Dendrobium venustum* and is abundant in northern, central and western Thailand. A previous study suggested that phoyunnanin E induces apoptosis in NSCLC cell lines via a p53-dependent pathway [223]. An in vitro study indicated that phoyunnanin E exhibits antimetastatic activity in H460 cells. Treatment of lung cancer cells with phoyunnanin E decreases the levels of active AKT/FAK, integrin-mediated migration, and the expression of EMT markers such as N-cadherin, vimentin, snail, and slug [224].

Curcumin, a bright yellow polyphenol-containing compound, is abundant in the species *Curcuma longa*. As a well-known natural product, curcumin possesses several pharmacological activities, such as antioxidant, anti-inflammatory, antidiabetic, antihypertension, and anticholesterol activities [225]. In lung cancer, curcumin suppresses cell proliferation by inhibiting PI3K/AKT signaling [226]. In addition to its antiproliferative activity, curcumin exhibits antimetastasis activity in NSCLC cell lines by reducing the levels of active AKT/mTOR [227].

In addition, sotetsuflavone, an active constituent of many traditional Chinese medicines, is present in several medicinal plants, especially in the *Cycas revoluta*. Sotetsuflavone is classified as a bioflavonoid, a polyphenol compound comprising two identical or nonidentical flavones connected to each other by an alkyl or an alkoxy-based linker of varying lengths [228]. This compound exerts anticancer effects on several cancer models, especially in lung cancer. Mechanistic investigations of sotetsuflavone in lung cancer have been reported; for example, it induces G0/G1 cell cycle arrest and cell death in A549 cells via a mitochondria-dependent pathway [229]. Its antimetastatic effect on A549 cells is mediated by the suppression of the HIF1α transcription factor and EMT through the PI3K/AKT and TNF-α/NF-κB signaling pathways [25], and it induces cell autophagy by downregulating the PI3K/AKT/mTOR pathway [230].

Luteoloside is also one of the active flavonoids detected in several medicinal herbs, especially in *Chrysanthemum morifolium*. Zhou and coworkers revealed that luteoloside inhibits lung cancer cell proliferation by inducing G0/G1 cell cycle arrest. A significant downregulation of p-AKT/p-mTOR/p-p70S6K was observed in response to luteoloside treatment. Moreover, luteoloside induces intracellular ROS formation associated with the suppression of the AKT/mTOR signaling pathway [231].

Cardamonin is a chalcone flavonoid that is abundant in *Boesenbergia rotunda*, one species in the *Zingiberaceae* family [232]. Its anticancer activities, such as apoptosis induction and metastasis suppression, occur through attenuation of the PI3K/AKT/mTOR pathway. Treatment with 20 µM cardamonin induces G2/M arrest and decreases the levels of EMT-related proteins. An in vivo H460 xenograft model showed significantly decreased levels of p-AKT and p-mTOR in the cardamonin group [233]. As a group of phenolics and flavonoids, its anticancer activity as an inhibitor of PI3K/AKT/mTOR signaling is mostly associated with its antioxidant capacity.

### 8.4. Quinoline

Quinoline is a group of molecules composed of heterocyclic aromatic rings that exhibit various pharmacological activities, such as antibacterial, antioxidant, and anticancer activities [222]. Jorunnamycin A is the natural marine tetrahydroisoquinoline isolated from Thai blue sponges (*Xestospongia sp.*). Jorunnamycin A shares a similar structure to the famous ecteinascidin 743 (trabectedin), which was approved by the FDA in 2015 as an anticancer drug targeting unresectable or metastatic liposarcoma [234]. According to Sirimangkalakitti et al., jorunnamycin A exhibits strong cytotoxicity toward NSCLC-H292 and H460 cells, with IC_50_ values of 220 and 160 nM, respectively [235]. Jorunnamycin A attenuates lung cancer cell migration by inhibiting AKT activity [236].

Likewise, renieramycin M is a novel tetrahydroisoquinoline natural marine compound that is isolated from a Thai blue sponge (*Xestospongia sp.*) [237]. Renieramycin M was reported to suppress cancer stem cell-like phenotypes [238]. Additionally, renieramycin M triggers lung cancer cell anoikis by decreasing AKT phosphorylation and downregulating prosurvival Bcl-2 family proteins [239,240]. The compounds in this group exert a strong inhibitory effect on AKT signaling at nanomolar concentrations and are promising candidates for research and development of drugs targeting lung cancer.

**Table 2:** Preclinical investigation of several natural products targeting the PI3K/AKT/mTOR signaling pathway in lung cancer.

Groups	Compound	Sources	Cell Lines	Mechanism of Actions	Refs.
Bibenzyl	4,5,4′-trihydroxy-3,3′-dimethoxybibenzyl (TDB)	*Dendrobium* *ellipsophyllum*	H292, H460 and H23	Induce apoptosis by downregulating AKT and upregulating p53 and proapoptotic proteins	[208]
			H292	Inhibit migration and invasion by downregulating AKT, FAK, CDC42 and integrins	[209]
			H292	Suppress metastasis by downregulating AKT and EMT signaling	[210]
	Gigantol	*Dendrobium draconis*	H460 and H292	Inhibit migration by downregulating AKT, CDC42 and Cav-1	[212]
			H460	Decrease the cancer stemness properties by inhibiting PI3K/AKT and JAK/STAT signaling	[213]
			H460	Downregulate active AKT, EMT markers and induce slug degradation	[214]
			H460	Sensitize cells to anoikis by suppressing the expression of AKT, ERK, Cav-1 and EMT markers	[215]
Phenanthrene	Ephemeranthol A	*Dendrobium* *infundibulum*	H460	Downregulate AKT, FAK and EMT markers	[218]
	Cypripedin	*Dendrobium* *densiflorum*	H460 and H23	Downregulate AKT and EMT markers	[220]
	Erianthridin	*Dendrobium* *formosum*	A549 and H460	Downregulate AKT/mTOR/p70S6K signaling	[96]
Phenolic and Flavonoid Compounds	Phoyunnanin E	*Dendrobium venustum*	H460	Inhibit migration by downregulating AKT and FAK signaling together with their downstream targets	[224]
	Curcumin	*Curcuma longa*	A549	Induce apoptosis and inhibit cell proliferation through the suppression of PI3K/AKT signaling and upregulation of miR-192-5p	[226]
			A549	Inhibit cell migration and invasion by decreasing PI3K/AKT/mTOR signaling and increasing miR-206	[227]
	Sotetsuflavone	*Cycas revolute*	A549	Induce autophagy by downregulating PI3K/AKT/mTOR signaling	[230]
			A549	Suppress the expression of HIF1α and its downstream targets, such as VEGF and MMPs, by downregulating PI3K/AKT and TNF-α/NF-κB	[25]
	Luteoloside	*Chrysanthemum morifolium*	A549 and H292	Induce cell cycle arrest and autophagy by inhibiting PI3K/AKT/mTOR/p70S6K signaling	[231]
	Cardamonin	*Boesenbergia* *rotunda*	H460, H1975, A549, H292, H1299 and HCC827	Inhibit proliferation and metastasis by downregulating the PI3K/Akt/mTOR pathway and its downstream targets	[233]
Quinoline	Jorunnamycin A	*Xestospongia* sp.	H460	Inhibition of AKT and EMT markers	[236]
	Renieramycin M	*Xestospongia* sp.	H460	Sensitize cells to anoikis by suppressing the expression of AKT and ERK, and downregulating Mcl-1 and Bcl-2	[239]

## 9. Conclusions and Future Perspectives

In this review, we summarized several drugs and natural compounds that target the PI3K/AKT/mTOR pathway in lung cancer. Thousands of species from plants, animals, and marine organisms have been investigated for their pharmacological activities. Both natural products and semisynthetic drugs are currently being investigated in preclinical and clinical trials. The majority of natural products that target this pathway and their downstream signaling intermediates, such as p70S6K, 4EBP1, and HIF1α, are associated with apoptosis and/or autophagy induction, suppression of the EMT, inhibition of migration and invasion and sensitization to chemotherapy. PI3K/AKT/mTOR inhibitors are designed to directly interact and inhibit these molecules.

According to accumulated studies of the PI3K/AKT/mTOR signaling pathway in lung cancer, this active signaling pathway clearly offers tremendous possibilities for therapies and challenges for drug research and discovery. However, targeted therapy acting on the PI3K/AKT/mTOR pathway may cause numerous side effects and defects due to the resistance acquired. The study of the specificity in cancer should be a serious concern, and the identification of novel dosing regimens that result in greater tolerance and overall efficiency of PI3K/AKT/mTOR inhibitors is required. Additional research should strive to overcome resistance to PI3K/AKT/mTOR inhibitors and suggest additional rational drug combinations.

## Data Availability

Not applicable.

## Sample Availability

Samples of the compounds presented in this review are not available from the authors.

## Abbreviations

ABD, adaptor binding domain; 4EBP1, 4E-binding protein 1; ALDH1A1, aldehyde dehydrogenase 1 family member 1A; ALK, anaplastic lymphoma kinase; ATP, Adenosine triphosphate; BAD, Bcl-2 associated agonist of cell death; Cav-1, caveolin-1; CD133, prominin-1; CDC42, cell division control protein 42 homolog; ECM, extracellular matrix; EGFR, Epidermal growth factor receptor; EMT, epithelial-mesenchymal transition; FAK, focal adhesion kinase; FKBP12, FK506-binding protein; FOXO3a, Forkhead box O3; GPCR, G-protein-coupled receptors; HIF-1α, Hypoxia-inducible factor-1α; HRE, hypoxia response element; MDM-2, Mouse double minute 2 homolog; mTOR, Mammalian target of rapamycin; MMPs, matrix metalloproteinases; NOS, nitric oxide synthase; NSAID, nonsteroidal anti-inflammatory drug; PDK1, phosphoinositide-dependent kinase-1; PHD, prolyl hydroxylase domain proteins; PI3K, Phosphatidylinositol-3-kinase; PIP2, phosphatidylinositol 4,5-bisphosphate; PIP3, phosphatidylinositol 3,4,5-trisphosphate; PKB/Akt, Protein kinase B; PTEN, phosphatase and tensin homolog; RBD, Ras-binding domain; RTK, receptor tyrosine kinase; S6K1, p70S6 kinase; SGK1, serine/threonine protein kinase 1; TDB, 4,5,4′-trihydroxy-3,3′-dimethoxybibenzyl; TRAIL, tumor necrosis factor-related apoptosis-inducing ligand; TSC1/2, tuberous sclerosis complex 1/2; VEGF, vascular endothelial growth factor; XIAP, X-linked inhibitor of apoptosis protein.
